# Significance of differential expression of thymidylate synthase in normal and primary tumor tissues from patients with colorectal cancer

**DOI:** 10.1186/1756-8722-4-33

**Published:** 2011-08-09

**Authors:** Yanyan Liu, Qingxin Xia, Yanzhao Jia, Hongqiang Guo, Bing Wei, Yawei Hua, Shujun Yang

**Affiliations:** 1 Department of Internal Medicine, Henan Provincial Cancer Hospital, Henan Provincial Institute of Cancer, Zhengzhou, Henan Province, China; 2 Department of Pathology, Henan Provincial Cancer Hospital, Henan Provincial Institute of Cancer, Zhengzhou, Henan Province, China; 3 Department of Surgery, Henan Provincial Cancer Hospital, Henan Provincial Institute of Cancer, Zhengzhou, Henan Province, China

## Abstract

The role of thymidylate synthase (TS) is essential as a key rate-limiting enzyme in DNA synthesis. It is the primary target of fluorouracil and its derivates in colorectal cancer. In this study, TS mRNA expression was examined in primary tumor and normal tissues from 76 patients with high- risk stage II/III colorectal cancer by laser capture microdissection and polymerase chain reaction. Thirty (39.47%) patients were found to have higher TS expression in primary tumors with earlier stage (P = 0.018), lower histological grades (P = 0.001) and high frequency microsatellite instability (P = 0.000). Multivariate analysis showed that microsatellite instability, histological grade and number of lymph nodes examined are independent prognostic markers.

## To the editor

Adjuvant chemotherapy can prolong survival of patients with high-risk, stage II/III colorectal cancer (CRC) after curative surgical resection [1-3]. Thymidylate synthase (TS) plays a major role as a key rate-limiting enzyme in DNA synthesis and serves as the primary target of fluorouracil and its derivates in CRC. It appears to have predictive and prognostic values [4,5]. Although the differences in TS expression among primary tumors, lymph nodes and other metastases have been reported [6], the pattern of TS expression in normal and tumor tissues has not been well characterized.

In this study, Laser capture microdissection (LCM) combined with polymerase chain reaction was used to examine TS gene expression in 76 patients with high risk II/III stage CRC (Table 1). Microsatellite instability (MSI, a panel of five loci recommended by National Cancer Institute) and clinicopathological features were analyzed at the same time.

**Table 1:** Clinicopathological Features

Features	Value
Number	76
Median age, year	58 (30-65)
Male sex, %	55.26
**Histological grades (%)**	
Low (G1 and G2)	75.00
High (G3 and G4)	25.00
**Tumor Site (%)**	46.8
Colon	27.63
Rectum	72.37
**Staging (%)**	
High-risk II	65.79
III	34.21
**Number of lymph node involved (%)**	
0	64.47
1-3	25.00
More than 3	11.00
**Median number of lymph node examined**	11 (6-38)
**Adjuvant regimen (%)**	
5-fluorouracil/leucovorin	86.84
5-fluorouracil/leucovorin/oxaliplatin	13.16

There were 32 (42.11%) patients with differential expression of TS gene between normal mucosa and primary tumor cells. Thirty of them (39.49%) have showed higher level of TS in primary tumors than in normal tissues.

To study the significance of the differential expression of TS in primary tumors, clinicopathological features, including age, gender, stage, histological grade, tumor site, and the number of lymph nodes examined, were analyzed and compared. The results showed that higher TS gene expression in primary tumors correlated to earlier stage (stage II, P = 0.018), and lower histological grades (G1 and G2, P = 0.001), but not to the other parameters defined above.

MSI was also examined in these patients. High frequency of MSI was found in 32.89% (25/76) of the patients in this study. The frequency of MSI was found to be significantly higher in patients with higher TS gene expression than those with lower TS expression (76.67% versus 4.35%, P = 0.000).

**Figure 1:**
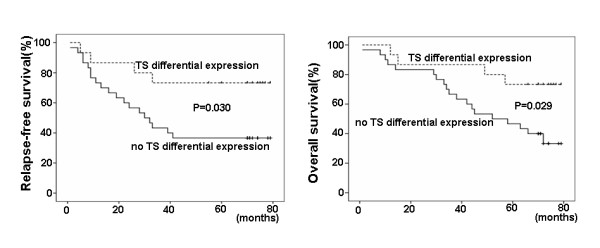
**Patients with higher TS gene expression in primary tumor cells had longer median relapse-free and overall survival (P = 0.030 and P = 0.029)**.

When the median time of relapse-free (RFS) and overall survival (OS) was compared, there was significant improvement of RFS (62.80 vs 40.67 months, p = 0.030) and OS (66.80 vs 51.67 months, p = 0.029) in patients with higher TS expression (Figure 1). Multivariate analysis was performed using a Cox regression model. MSI (P = 0.034), histological grade (P = 0.001) and the number of lymph nodes examined (P = 0.014) were identified as independent prognostic markers in this study.

In summary, patients with higher TS expression in the primary tumors, high frequency MSI, lower histological grades and more than 10 lymph nodes examined had better outcome after adjuvant chemotherapy.

## List of Abbreviations

CRC: Colorectal cancer; TS: thymidylate synthase; LCM: Laser capture microdissection; MSI: Microsatellite instability.

## Authors' contributions

YL and SY designed the study, interpreted data; YL wrote the manuscript; QX and BW reviewed diagnoses of pathology; YJ performed the experiment; HG performed the statistical analysis; YH collected patient data and samples.

All authors have read and approved the final manuscript.

## Conflict of interests

The authors declare that they have no competing interests.
